# High Performance of Mn_2_O_3_ Electrodes for Hydrogen Evolution Using Natural Bischofite Salt from Atacama Desert: A Novel Application for Solar Saline Water Splitting

**DOI:** 10.3390/ma17205129

**Published:** 2024-10-21

**Authors:** Felipe M. Galleguillos-Madrid, Sebastian Salazar-Avalos, Edward Fuentealba, Susana Leiva-Guajardo, Luis Cáceres, Carlos Portillo, Felipe Sepúlveda, Iván Brito, José Ángel Cobos-Murcia, Omar F. Rojas-Moreno, Víctor Jimenez-Arevalo, Eduardo Schott, Alvaro Soliz

**Affiliations:** 1 Centro de Desarrollo Energético Antofagasta, Universidad de Antofagasta, Antofagasta 1240000, Chile; sebastian.salazar@uantof.cl (S.S.-A.); edward.fuentealba@uantof.cl (E.F.); susana.leiva.guajardo@ua.cl (S.L.-G.); carlos.portillo@uantof.cl (C.P.); 2 Departamento de Ingeniería Química y Procesos de Minerales, Universidad de Antofagasta, Av. Universidad de Antofagasta 02800, Antofagasta 1271155, Chile; luis.caceres@uantof.cl; 3 Departamento de Ingeniería en Minas, Universidad de Antofagasta, Antofagasta 1240000, Chile; felipe.sepulveda@uantof.cl; 4 Departamento de Química, Facultad de Ciencias Básicas, Universidad de Antofagasta, Antofagasta 1240000, Chile; ivan.brito@uantof.cl; 5 Instituto de Ciencias Básicas e Ingeniería, Universidad Autónoma del Estado de Hidalgo, Carr. Pachuca—Tulancingo km. 4.5, Mineral de la Reforma, Hidalgo C.P. 42184, Mexico; jose_cobos@uaeh.edu.mx; 6 Faculty of Mechanic, Electronic and Biomedical Engineering, Universidad Antonio Nariño, Tunja 150002, Colombia; omar.rojas@uan.edu.co; 7 Departamento de Química de los Materiales, Facultad de Química y Biología, Universidad de Santiago de Chile, Av. Libertador B. O’Higgins 3363, Santiago 9170022, Chile; victor.jimenez@usach.cl; 8 Departamento de Química Inorgánica, Facultad de Química y Farmacia, Centro de Energía UC, Centro de Investigación en Nanotecnología y Materiales Avanzados CIEN-UC, Pontificia Universidad Católica de Chile, Avenida Vicuña Mackenna, 4860, Santiago 7820436, Chile; edschott@uc.cl; 9 Departamento de Ingeniería en Metalurgia, Universidad de Atacama, Av. Copayapu 485, Copiapó 1530000, Chile

**Keywords:** saline water splitting, hydrogen evolution reaction (HER), oxygen reduction reaction (ORR), bischofite mineral, mixed potential theory

## Abstract

Solar saline water splitting is a promising approach to sustainable hydrogen production, harnessing abundant solar energy and the availability of brine resources, especially in the Atacama Desert. Bischofite salt (MgCl_2_·6H_2_O) has garnered significant attention due to its wide range of industrial applications. Efficient hydrogen production in arid or hyper arid locations using bischofite solutions is a novel and revolutionary idea. This work studied the electrochemical performance of Mn_2_O_3_ electrodes using a superposition model based on mixed potential theory and evaluated the superficial performance of this electrode in contact with a 0.5 M bischofite salt solution focusing on the hydrogen evolution reaction (HER) and oxygen reduction reaction (ORR) that occur during saline water splitting. The application of the non-linear superposition model provides valuable electrochemical kinetic parameters that complement the understanding of Mn_2_O_3_, this being one of the novelties of this work.

## 1. Introduction

The Atacama Desert contains substantial deposits of bischofite mineral salt (MgCl_2_·6H_2_O), a form of magnesium chloride that forms during the solar evaporation process used for lithium recovery. Typically, during the recovery of saline sediments from the Atacama Salar, natural brine undergoes solar evaporation until a concentration of 30% *w*/*w* LiCl is achieved, after the formation of halite, sylvite, carnallite, and bischofite [1]. Bischofite is notably abundant in the Atacama Desert, primarily found in brines located in high-altitude regions near the Andes Mountains.

The electrochemical stability of electrodes in the water splitting process is essential for ensuring the long-term efficiency and effectiveness of HER-based energy systems. Other oxides with different valence states, such as Mn_3_O_4_ and Mn_2_O_3_, are also possible cathodic material candidates. Mn_2_O_3_ has generated interest because of its high theoretical specific capacity and energy density as α-Mn_2_O_3_ between 500 and 800 °C [2] and in forming ternary oxides as Mn_2_O_3_ [3,4,5]. The family of MnO_2_ is characterized by a variety of polymorphs with both open (with tunnels) and laminar structures such as hollandite, todorokite, and birnessite [6]. Mn_2_O_3_ powder offers significant advantages in terms of cost-effectiveness and abundance compared to traditional noble metal catalysts, where the use of abundant and affordable materials for HER catalysis is essential for promoting large-scale hydrogen production and making it economically viable. Mn_2_O_3_ powder presents an opportunity to address the cost and availability concerns associated with noble metal catalysts, thereby accelerating the adoption of hydrogen as a clean and sustainable energy carrier. The interaction between the Mg and Mn powder plays a significant role in the HER activity and selectivity during the cathodic subprocess. Understanding the structural aspects of Mn_2_O_3_ powder and its correlation with catalytic performances is crucial for further optimizing the properties and tailoring it for specific applications. Furthermore, Mn_2_O_3_ powder demonstrates excellent performance not only in the HER but also in other important electrochemical reactions, such as the oxygen evolution reaction (OER) and oxygen reduction reaction (ORR), respectively [7]. However, there is currently a lack of plausible models that accurately describe the electrochemical and oxidation parameters during the mechanisms. Its versatility and multifunctionality make it a valuable candidate for integration into various renewable energy conversion and storage devices, including water electrolyzers and fuel cells. The ability to perform multiple electrochemical reactions with high efficiency further highlights the potential of Mn_2_O_3_ powder as a versatile catalyst for clean energy technologies.

This paper presents pioneer insights into the utilization of Mn_2_O_3_ powder as an electrocatalyst material for the HER and ORR mechanisms using bischofite mineral recovery from the solar evaporation process present in the Atacama Desert. The principal novelty of using bischofite solution for saline electrolysis, particularly in the context of hydrogen evolution reactions (HERs), stems from several distinctive properties and benefits that this mineral-rich solution offers compared to traditional electrolytes like pure magnesium chloride solutions. This was performed in an artificial 0.5 M MgCl_2_ and 0.5 M of natural bischofite solution compared with 0.5 M NaCl using a superposition model based on mixed potential theory and determination of the electrochemical and kinetic parameters of the HER, ORR, and MnOR reactions. Additionally, a comprehensive surface analysis was conducted using scanning electron microscopy (SEM), X-ray diffraction (DRX), and energy-dispersive X-ray spectroscopy (EDS) imaging techniques.

## 2. Materials and Methods

### 2.1. Synthesis of Mn_2_O_3_

The active material Mn_2_O_3_ was prepared by means of the co-precipitation method from a solution containing MgSO_4_ and MnSO_4_·H_2_O (Sigma-Aldrich, 99% purity, St. Louis, MO, USA) with a ratio of 1:2. The solution contained dissolved 1 M NaOH (Sigma-Aldrich, 99% purity, St. Louis, MO, USA) to keep the alkaline media (pH ~ 8) and distilled water with 18.0 mΩ/cm of resistivity. The solution was kept under magnetic stirring (500 rpm) for 20 h to complete the reaction. The product of this reaction was a pink solid powder, which was subsequently filtered and dried at 80 °C for 36 h. This last obtained powder was washed 5 times with plenty of deionized water to eliminate the remaining Na^+^ and part of SO_4_^2−^ ion remnants present in the precipitate obtained after the reaction. Once the solid was clean, it was introduced into a furnace at a temperature of 800 °C for 6 h to form the Mn_2_O_3_ material and then allowed to cool to room temperature. The Mg was incorporated into the Mn_2_O_3_ dispersed over the spinel.

### 2.2. Electrochemical Measurements of Mn_2_O_3_ Electrode

The electrochemical measurement procedure was designed to examine the kinetics of the partial electrochemical reactions on the Mn_2_O_3_ spinel electrode immersed in 0.5 M NaCl, 0.5 M MgCl_2_, and 0.5 M of natural bischofite solution, focusing the attention on the HER, ORR, and MnOR (Mn oxidation reaction). The bischofite salt was collected from stockpile from solar evaporation process in the Atacama Salar (see graphical abstract), the samples were storage in sealed beaker and maintained at room temperature. Considering that the mineral is mainly composed by MgCl_2_·6H_2_O (as is shown in the XRD and EDS analysis) and impurities are Na^+^ and K^+^, respectively. The tests solutions were prepared by direct weighting of the mineral and dissolved in deionized water at a 0.5 M concentration.

A modified carbon paste electrode was prepared by mixing 0.2 g of Mn_2_O_3_ powder, 0.25 g of graphite powder, and 0.2 cm^3^ of paraffin wax up to obtain a homogeneous paste. This paste was tightly packed into a plastic Teflon sheath (4 mm in diameter and 10 mm in length) that had adequate perforations to maintain electrical contact through a copper wire and the Mn_2_O_3_ powder with the rotating disc electrode (RDE) system. Current density vs. potential curves were obtained by linear sweep voltammetry (LSV) measurements using a BASI/RDE-2 rotating electrode interphase (Basi Corp. West Lafayette, IN, USA) connected to an Epsilon potentiostat/galvanostat (Basi Corp. West Lafayette, IN, USA) in a conventional 3-electrode cell, in which the carbon paste electrode with Mn_2_O_3_ powder acted as the working electrode (WE), a platinum wire acted as the counter electrode (CE), and Ag/AgCl (4M KCl Sat.) acted as a reference electrode (RE). All potentials reported are referred to as the standard hydrogen electrode (SHE). The experimental protocol for polarization data was according to a previous work [8] in a potential range between −1000 and 400 mV/SHE. Before each run, the Mn_2_O_3_ spinel electrode was maintained at −1000 mV/SHE for 30 s.

### 2.3. Kinetic Analysis

The kinetic analysis was performed by applying non-linear fitting to experimental polarization curves obtained from LSV measurements. The superposition model based on the mixed potential theory was considered according to the methodology described in our previous work concerning mass diffusion, charge transfer, and oxidation mechanism controls [9,10,11]. The following set of kinetic expressions (Equations (1)–(4)) were used as part of the non-linear fitting methodology, which considers the total current density (i) as a function of the partial current densities for the ORR ($i_{O_{2}}$), HER ($i_{H_{2}}$), and MnOR ($i_{Mn}$):

(1) $$i=i_{H_{2}}+i_{O_{2}}+i_{Mn}$$

(2) $$i_{O_{2}}=i_{0,O_{2}}{\left(\frac{i_{l,O_{2}}-i_{O_{2}}}{i_{l,O_{2}}}\right)}^{m}\cdot e^{\left(\frac{2.303\eta_{O_{2}}}{t_{O_{2}}}\right)}$$

(3) $$i_{H_{2}}=i_{0,H_{2}}\cdot e^{\left(\frac{2.303\eta_{H_{2}}}{t_{H_{2}}}\right)}$$

(4) $$i_{Mn}=i_{0,Mn}\cdot e^{\left(\frac{2.303\eta_{Mn}}{t_{Mn}}\right)}$$

where $i_{0{,O}_{2}}$, $i_{0,H_{2}}$, and $i_{0,Mn}$ are the exchange current densities for the ORR, HER, and MnOR, respectively. $i_{l,O_{2}}$ is the limiting current density for the ORR. $\eta_{O_{2}}=E-E_{eq,O_{2}}$, $\eta_{H_{2}}=E-E_{eq,H_{2}}$, and $\eta_{Mn}=E-E_{eq,Mn}$ are the overpotentials for the ORR, HER, and MnOR, respectively. E is the electrochemical applied potential, and $E_{eq,O_{2}}$, $E_{eq,H_{2}}$, and $E_{eq,Mn}$ are the equilibrium potentials for the ORR, HER, and MnOR, respectively. $t_{O_{2}}$, $t_{H_{2}}$, and $t_{Mn}$ are Tafel slopes for the ORR, HER, and MnOR, respectively, and m is the kinetic order for the ORR and is equal to 1. The kinetic parameters were calculated from the fitting of Equations (1)–(4) to the experimental data.

### 2.4. Mn_2_O_3_ Electrode Surface Characterization

The morphological characterization of the samples was performed by energy dispersive X-ray spectroscopy (EDS) using the following instruments: a Zeiss EVO MA 10 (Zeiss, Oberkochen, Germany) and a SEM Hitachi model SU-5000 (Tokyo, Japan). The products layer was studied by X-ray diffraction (XRD) in a Shimadzu XRD-600 diffractometer (Shimadzu Corp., Kyoto, Japan) using Cu Kα radiation at an angular step of 0.02° (2θ) and counting time per step of 4 s.

## 3. Results

**Figure 1:**
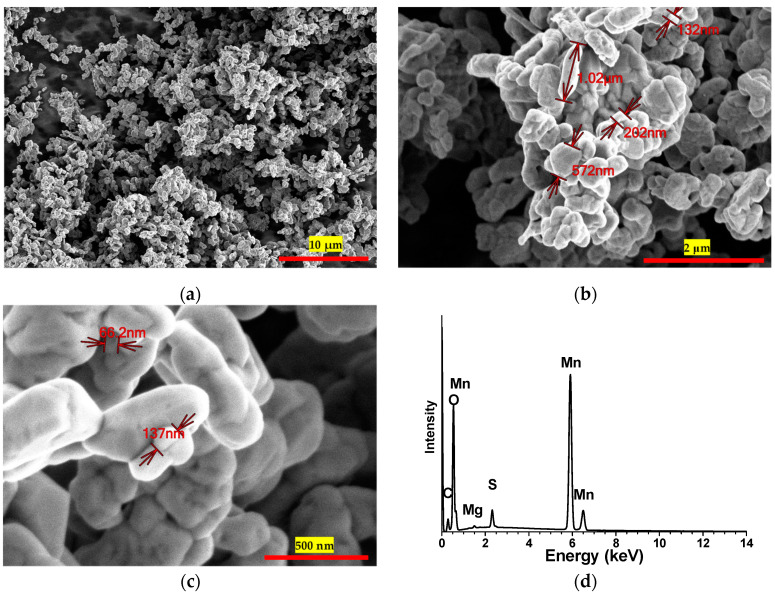
SEM images depicting the synthesized Mn_2_O_3_ material, captured at varying magnifications: (**a**) 3000×, (**b**) 20,000×, and (**c**) 70,000×. Additionally, (**d**) presents the corresponding EDS spectrum obtained from the sample.

Figure 1 shows a sequence of scanning electron microscopy (SEM) images portraying the synthesized Mn_2_O_3_ material. These images were captured at varying magnifications: 3000×, 20,000×, and 70,000×. Accompanying these images is the EDS spectrum extracted from the sample. These micrographs offer a comprehensive view of the material morphology and structure, highlighting the characteristics of the granules present within the sample. Notably, the Mn_2_O_3_ powder exhibits a self-limiting growth pattern. The uniformity in their size is readily apparent; the average kernel size was determined to be 598, with a standard deviation of approximately 334.33. While this standard deviation signifies the presence of granules spanning diverse sizes, it is evident that most of these granules tend to cluster around the mentioned average size.

**Figure 2:**
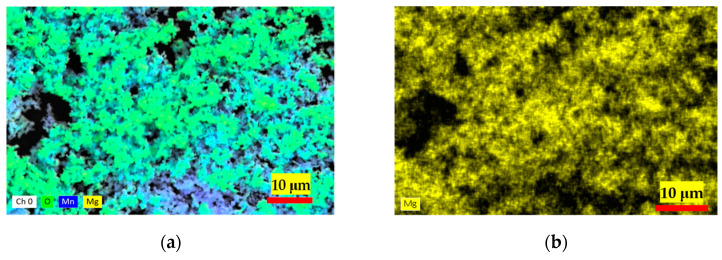

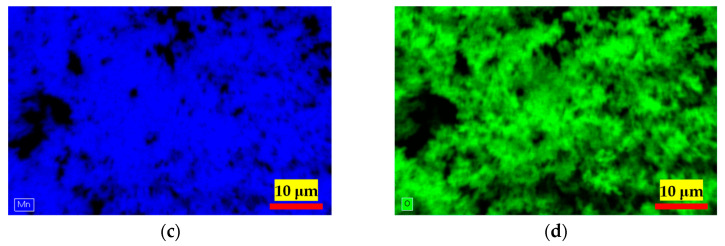
Elemental mapping by EDS analysis for Mn_2_O_3_ powder. (**a**) Multi-elemental distribution of Mn, Mg, and O elements; (**b**) Mg distribution; (**c**) Mn distribution; and (**d**) O distribution.

Moreover, the EDS spectrum (Figure 2) acquired by utilizing secondary electrons with an energy of 30 keV furnishes an intricate depiction of the elemental constitution of the material. This EDS analysis revealed a composition of 57.53 ± 2.7% manganese (Mn), 31.29 ± 10.36% oxygen (O), and 2.1 ± 4.82% sulfur (S) as impurities into the material. Considering the anticipated composition of Mn_2_O_3_, we antedated a manganese (Mn) weight percentage of 69.6% and an oxygen (O) weight percentage of 30.4%. When contrasting these anticipated values with our EDS findings, deviations became evident. The EDS assessment showed a manganese (Mn) proportion of 57.53 ± 2.7%, slightly lower than the projected theoretical value. Correspondingly, the oxygen (O) content measured at 31.29 ± 10.36% was marginally higher, yet this discrepancy fell within the range accounted for by the standard deviation, relative to the theoretical estimate. Numerous factors might contribute to this disparity, ranging from the inherent nature of the synthesis process, which could yield a non-pure Mn_2_O_3_ product, to potential anomalies or interferences encountered during the EDS analysis. These values underscore that while the sample predominantly comprises Mn and O, aligning with the Mn_2_O_3_ expectation, the presence of S, although in a diminished ratio, raises the prospect of a potential impurity originating from the raw materials employed in the synthesis process. This insight leads to the inference that the synthesis of Mn_2_O_3_ was achieved at a noteworthy level of purity.

The Inclusion of Mn^3+^ within the Mn_2_O_3_ structure introduces specific structural distortions that stem from the Jahn–Teller effect. This effect, inherent in certain cations with distinct electronic configurations, can induce asymmetries within the crystalline arrangement. In the context of Mn_2_O_3_, these distortions hold the potential to exert influence over the physical material and chemical attributes. The Mn_2_O_3_ exhibits a propensity for thermal transformations. Notably, under temperatures nearing 550 °C, noteworthy alterations in the tetragonal distortion of the spinel structure can arise. This phenomenon is closely intertwined with the Jahn–Teller effect and the concurrent presence of Mn^3+^ within the structure, signifying a connection between electronic interactions and structural changes.

Through meticulous analysis and comprehensive deliberation of the scanning electron microscopy (SEM) and X-ray diffraction (XRD) findings, an unequivocal validation of the successful Mn_2_O_3_ formation via the employed synthesis process was attained. The SEM images, in conjunction with the granule morphology characterization and the illustration of their self-limiting growth, furnish compelling substantiation of the synthesized Mn_2_O_3_’s inherent nature and purity. Concurrently, the discernible XRD pattern, showcasing distinctive peaks emblematic of the crystalline Mn_2_O_3_ structure, reasserts the compound’s presence and precise crystalline configuration.

**Figure 3:**
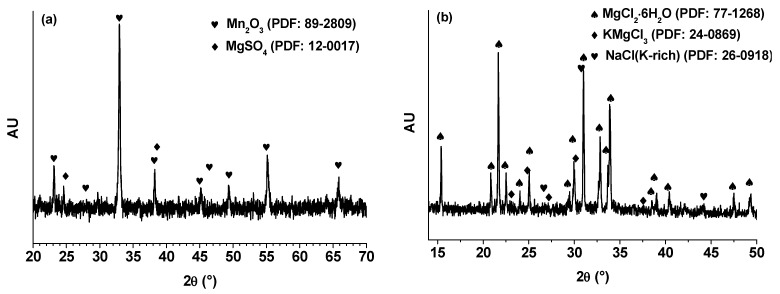
(**a**) XRD pattern of Mn_2_O_3_ powder showing a characteristic peak at (h, k, l) = 2, 2, 2 with a 2θ angle of 32.954°, and (**b**) XRD pattern of bischofite showing a characteristic peak at (h, k, l) = 2 2, 2 with a 2θ angle of 16.32°.

The results derived from the X-ray diffraction (XRD) analysis of the Mn_2_O_3_ powder are shown in Figure 3a. Particularly, a discrete peak is distinguished at the 2θ angle of 32.954°, positioning with a crystalline plane of (h, k, l) = 2, 2, 2 and displaying a relative intensity of 100%. This prominent peak serves as a definitive marker for the distinctive crystalline arrangement of Mn_2_O_3_. By XRD analysis, pivotal insights into the crystalline structure of Mn_2_O_3_ come to light.

At the same time, the results obtained from the XRD analysis of the natural bischofite (MgCl_2_·6H_2_O) salt are presented in Figure 3b, in which a dissimilar peak appears at the 2θ angle close to 16.32°, corresponding to the crystalline plane with indices of (h, k, l) = 0, 0, 1 and showing a relative intensity of 100%. This peak serves as a clear indicator of the unique crystalline structure of bischofite. However, the natural bischofite has particular impurities such as K^+^ and Na^+^ from carnallite and halite salts present in the composition of well brine. The XRD analysis reveals crucial details about the crystalline arrangement of bischofite.

The combination of these analytical techniques strengthens our confidence in the acquired outcomes, affirming the unequivocal attainment of Mn_2_O_3_ formation throughout the synthesis effort. This assurance assumes paramount significance, as the purity and accurate formation of Mn_2_O_3_ wield direct influence over its properties and potential applications within both research and industrial contexts.

**Figure 4:**
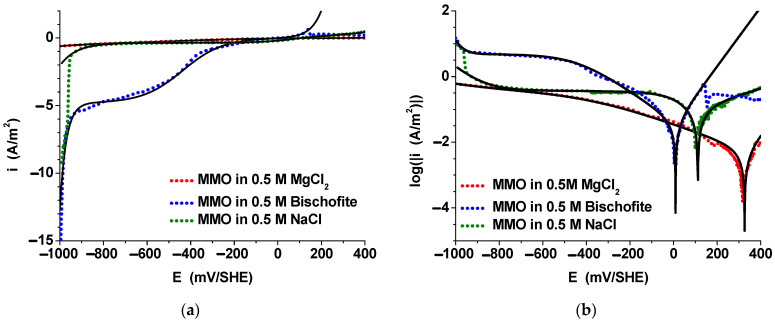
Electrochemical performance of Mn_2_O_3_ electrode. (**a**) Linear polarization curves, (**b**) Tafel polarization curves, and superposition model output curves for the Mn_2_O_3_ electrode in aerated 0.5 M NaCl (green line), 0.5 M MgCl_2_ (red line), and 0.5 M bischofite (blue line) at 2 mV/s and 600 RPM, assuming a kinetic order of 1 for the ORR. The black line represents the model fitting according to mixed potential theory.

The kinetic study was accomplished by applying non-linear fitting to experimental polarization data, considering the superposition model and mixed potential theory according to the methodology described in our previous works in terms of charge transfer, mass diffusion, and oxidation mechanism controls [8,11]. Figure 4 provides important information about the electrochemical–kinetic performance of Mn_2_O_3_ powder during the sub-cathodic process in contact with 0.5 M NaCl, 0.5 M MgCl_2_, and 0.5 M MgCl_2_·6H_2_O solutions. Figure 4a displays the linear sweep voltammetry (LSV) results, where the potential window applied during the experiments ranges from −1000 to 400 mV/SHE.

When comparing the performance of Mn_2_O_3_, where H_2_ evolution is predominant, using the bischofite solution shows a slightly higher variation compared to using pure MgCl_2_ solution during the HER mechanism. This could be due to the presence of natural impurities in the bischofite that might act as co-factors promoting the charge transfer process. As for the cathodic potential range where the hydrogen evolution reaction (HER) mechanism occurs, in the range of −1000 to −800 mV, the Tafel slope ($t_{H_{2}}$) was about −172, −471, and −68 mV/dec for 0.5 M NaCl, 0.5 M MgCl_2_, and MgCl_2_·6H_2_O.

## 4. Discussion

The comparative analysis of electrochemical parameters across three different electrolytes (0.5 M MgCl_2_, 0.5 M bischofite, and 0.5 M NaCl) provides insights into their varying electrochemical behaviors. The $i_{0,H_{2}}$ for Mn_2_O_3_ is significantly higher when in contact with 0.5 M MgCl_2_ compared to natural 0.5 M bischofite or 0.5 M NaCl. However, the t_H2_ of bischofite demonstrates a much lower Tafel slope, suggesting faster kinetic processes for the HER, and a substantially higher $i_{l,O_{2}}$ indicating better performance under high-current conditions relative to 0.5 M MgCl_2_ and 0.5 M NaCl (refer to Table 1).

During the ORR, $i_{l,O_{2}}$ was −0.370, −0.351, and −4.810 A/m^2^ for NaCl, MgCl_2_, and natural bischofite, respectively. For natural bischofite, the higher value of $i_{l,O_{2}}$ indicates limitations in the mass transport, suggesting better performance in facilitating the oxygen-related process. The $i_{0,O_{2}}$ for both electrolytes was negligible, which implies that the intrinsic rate of the oxygen-related electrochemical reaction was too low in both cases. These low $i_{0,O_{2}}$ values suggest that the kinetics are slow at equilibrium [7,12,13]. In the case of NaCl, its $i_{0,O_{2}}$ was similar to that of bischofite (see Table 1).

The resistance of the Mn_2_O_3_ powder electrode in contact with pure MgCl_2_ and the natural bischofite solution yielded $E_{corr}$ and $i_{corr}$ with values of 324 and 9 mV/SHE and 5.66 × 10^−3^ and 3.89 × 10^−2^ A/m^2^, respectively. The $E_{corr}$ and $i_{corr}$ values indicate a high resistance of corrosion during the HER mechanism, which is supported by the performance of the Evans curve shown in Figure 4b and the electrochemical kinetic parameters for the HER, ORR, and MnOR tabulated in Table 1.

Table 1 presents the key corrosion and kinetic parameters obtained from the application of the superposition model, utilizing Equations (1)–(4). The parameters are presented in relation to their respective controls, including charge transfer, mass diffusion, and dissolution mechanisms. This comprehensive analysis provides valuable insights into the governing factors and mechanisms influencing the corrosion behavior in the studied system.

The Tafel slope for the HER using Mn_2_O_3_ in bischofite solution was −68 mV/dec, which indicates that there exists a stronger linear relationship between the logarithm of the current density and the overpotential, with a negative slope representing a decrease in overpotential as the current density increases. Meanwhile, the Tafel slope for the MgCl_2_ solution was −471 mV/dec, which suggests efficient HER kinetics. The variations in the kinetic and corrosion parameters could be the product of impurities like Na^+^ and K^+^, respectively. Impurities in bischofite, such as K^+^ and Na^+^, increase the conductivity of the solution by contributing additional charge carriers. Higher conductivity generally enhances the efficiency of electrochemical processes by reducing the resistance of the electrolyte, potentially increasing the rate of electron transfer and reducing the energy required for reactions. Additionally, K^+^ and Na^+^ ions influence the overpotential necessary to initiate electrochemical reactions and help to stabilize intermediate species during the HER or ORR, thereby reducing the overpotential needed for these reactions to proceed. On the other hand, K^+^ and Na^+^ ions are less likely to adsorb strongly onto surfaces compared to multivalent cations such as Mg^2+^. However, the presence of K^+^ and Na^+^ affects the rate of charge transfer reactions, including corrosion processes. This enhanced conductivity could lead to an increase in $i_{corr}$ to about 9 A/m^2^, whereas MgCl_2_ and NaCl had values of 324 and 112 mV/SHE, respectively, indicating a higher rate of corrosion under experimental conditions. These ions can interact with the metal surface or corrosion products, potentially forming complexes or altering the protective nature of any passive layers. If these interactions weaken the passive layer, the corrosion rate could increase, as reflected by a higher $i_{corr}$. In terms of $E_{corr}$, these ions shift the balance towards more oxidative conditions or positive potentials, creating a more reducing environment, which might shift to more negative values (see Table 1).

Numerous electrode materials have shown promise for the HER mechanism in seawater or brine. For these materials, it is important to note that the H_2_ Tafel slope represents the rate of the HER and depends on specific experimental conditions and electrode preparation methods [14,15]. In seawater, the Pt is often considered the standard for the HER due to its excellent catalytic activity with a Tafel slope that typically ranges from around −30 to −40 mV/dec [16,17]. The Tafel slope of Ni-Fe alloys fluctuates from −91 to −149 mV/dec and depends on the specific Ni-Fe alloy composition and the surface modifications [18,19]. However, Ni-Mo has Tafel slopes in the range of −40 to −60 mV/dec. The Tafel slope for the CoP material is typically between −40 and −50 mV/dec in 1 M KOH [20,21,22,23]. Other studies are related to water saline splitting [24,25,26,27,28].

## 5. Conclusions

The use of Mn_2_O_3_ powder as an electrode for hydrogen evolution reactions (HERs) in contact with solutions of NaCl, pure MgCl_2_, and natural bischofite salt shows significant differences in electrochemical performance and kinetic parameters determined using non-linear fitting, which proved to be an efficient analysis tool. This superposition model, based on mixed potential theory, effectively described the controls of mass diffusion, charge transfer, and oxidation mechanisms during saline water splitting for all electrolytes and can predict the performance of Mn_2_O_3_ powder in contact with other electrolytes. The Tafel slopes from Evans curves indicate that natural bischofite salt solution is more efficient for the HER mechanism, characterized by a Tafel slope of −68 mV/dec compared to −471 and −172 mV/dec for pure 0.5 M MgCl_2_ and 0.5 M NaCl, respectively. This lower Tafel slope for bischofite solution suggests faster kinetics and higher efficiency for H_2_ evolution, and so a smaller increase in overpotential to achieve higher current densities could be required for effective charge transfer processes and a better catalytic performance. Concerning the limiting current densities ($i_{l,O_{2}}$) for the electrolytes, the values were 0.351 and 4.810 A/m^2^ for pure MgCl_2_ and for natural bischofite salt, respectively. The significant increase in the absolute value of the limiting current density for the natural bischofite shows its superior performance in supporting faster oxygen-related processes before mass transport limitations occur. However, the low exchange current densities ($i_{0,O_{2}}$) for both electrolytes indicate slow intrinsic reaction kinetics at equilibrium, highlighting a common limitation in both systems. The use of bischofite salt from Atacama Salar as an electrolyte in electrolysis for H_2_ production is an innovative approach, especially relevant in the context of renewable energy and the use of local resources in the Atacama Desert.

## Figures and Tables

**Table 1:** Kinetic and corrosion parameters calculated from experimental polarization curves for Mn_2_O_3_ steel immersed in NaCl, MgCl_2_, and bischofite solutions using the superposition model according to mixed potential theory.

Parameters	0.5 M MgCl_2_	0.5 M Bischofite	0.5 M NaCl
$i_{0,H_{2}}$, A/m^2^	−1.40 × 10^−2^	−9.85 × 10^−9^	−5.17 × 10^−4^
$t_{H_{2}}$, mV/dec	−471	−68	−172
$i_{0,O_{2}}$, A/m^2^	−3.12 × 10^−4^	−5.78 × 10^−6^	−3.21 × 10^−6^
$t_{O_{2}}$, mV/dec	−400	−213	−151
$i_{l,O_{2}}$, A/m^2^	−0.351	−4.810	−0.37
$i_{0,Mn}$, A/m^2^	2.61 × 10^−8^	3.45 × 10^−6^	9.72 × 10^−3^
$t_{Mn}$, mV/dec	143	111	511
$E_{corr}$, mV/SHE	324	9	112
$i_{corr}$, A/m^2^	5.66 × 10^−3^	3.89 × 10^−2^	0.12

## Data Availability

The raw data supporting the conclusions of this article will be made available by the authors on request.
